# Phenotypic Screens, Chemical Genomics, and Antimalarial Lead Discovery

**DOI:** 10.1371/journal.ppat.1002156

**Published:** 2011-08-18

**Authors:** Jose F. Garcia-Bustos, Francisco-Javier Gamo

**Affiliations:** Tres Cantos Medicine Development Campus, Diseases of the Developing World, GlaxoSmithKline, Tres Cantos, Spain; The Fox Chase Cancer Center, United States of America

Several extensive small molecule screens against growing *Plasmodium falciparum* have recently been published [1]–[3] and thousands of hit structures are now publicly available. This represents a large majority of the drug-like chemical diversity currently available for screening and hence delineates the currently drugable target space of *P. falciparum*, since the “drugability” term includes an “availability” concept. From a chemical standpoint, some hits may look like bona fide drug leads while others more like chemical probes for target identification, but if the hit set is globally biased for any physicochemical property, relative to the starting screening libraries, it is the microorganism that “selected” for it. We can ask next what is the nature of the bias, and whether the chemical diversity identified in the screens is a reasonable representation of the chemotypes needed to inhibit the essential and potentially drugable targets in the pathogen. The usual answer to that question is “surely not”, but why?

The starting compound libraries are purposely biased to fit into the “ADME space” for orally bioavailable compounds [4], [5] and by the practicalities of synthetic chemistry. Screening libraries at companies also reflect their interest in certain human targets, although in GlaxoSmithKline's case half of the starting compounds were purchased from outside vendors, and other published hit sets contain commercial compounds only [2], [3]. It is difficult to estimate what coverage of target space has been achieved with the published structures. Nobody knows the total number of potentially drugable targets in *Plasmodium*, but as a first approximation we can use the figure of 400 predicted eukaryotic core essential genes [6]. Some gene functions will not be drugable, but others not belonging to the core set may be indispensable in the human host cell.

In principle we could use the chemical families identified in the screens to roughly estimate the number of therapeutically relevant targets, meaning those that can be lethally affected by achievable concentrations of drug-like compounds. However, in the authors' view, establishing a one-to-one correlation would be unsatisfactory, as we would need to assume that each chemical family inhibits a different target. Chemoinformatic tools to classify compounds leave plenty of room to make each classification subjective. Chemists accept as a fact of life that the same compound can be classified in either one of two, or even more, related chemical families. That may carry less consequence in terms of chemical thinking, but in the context of this discussion, it means we cannot reliably establish a univocal correspondence between chemical families and individual targets. During our ongoing analysis of the Tres Cantos Antimalarial Set (TCAMS) [1] (deposited at https://www.ebi.ac.uk/chemblntd together with similar sets from St. Jude Children's Research Hospital and Novartis-GNF), we are finding that compounds in the same chemical family show different parasitological properties. Some inhibit an identified essential enzyme while others do not, and some exhibit a delayed-death phenotype, but not so their fellow class members. Conversely, different chemical families are being found to kill parasites through inhibition of the same target. These findings show that a one chemical family–one target correlation cannot be reliably established when based on purely chemical criteria.

Computational tools to analyze hit sets need to be biologically informed in order to be useful for generating target hypotheses. It is not helpful simply trying to define “the” physicochemical properties common to antimalarial hits from whole-cell screens. One would not expect all binding sites for small ligands in a microorganism to have common features, and that they will differ between taxonomic divisions. So unless there is one, or very few, prevailing killing mechanism for most compounds in the set, the dominant requirements common to all hits against a given pathogen will be those broadly related to cellular transport (influx, efflux, and intracellular accumulation). Biological information must be layered on top of the chemical clustering to make it useful for investigating the target space of a pathogen. Computational exercises can estimate chemical similarities between compounds in the set and known ligands of specific proteins, or estimate the physicochemical complementarity between compounds and binding sites in proteins with known or modelled structures. Given the large gaps in the basic knowledge of *Plasmodium*, all these analyses require a great deal of extrapolation and lots of modelling, anchoring target predictions to very few known structures and making them highly operator dependent. The approach has recently claimed some successes [7]–[9], but to date most practitioners admit that truly novel insights are usually needles in a haystack of already known or strongly suspected targets.

Sets of whole-cell hits should be re-screened for specific modes of action. Even with the small numbers of compounds in such sets, single target screens are probably not practical given the effort required to validate individual targets and develop robust assays, and attempts in that direction have failed to produce useful results so far [10]. But perhaps the scientific community could come up with broad triaging assays, somewhat parallel to what the yeast community undertook to find functions for genes in the then newly sequenced genome of *Saccharomyces cerevisiae* [11]. They collectively generated and exchanged well-defined mutant strains that were tested in simple assays to cluster the affected genes into broad phenotypic groups. TCAMS is being screened against *P. falciparum* under in vitro conditions that make the parasite resistant to inhibition of certain metabolic pathways, allowing identification of groups of compounds inhibiting any target in those pathways (unpublished data). But the malaria community has developed or is developing wider and more informative assays able to detect interference with complex processes, such as protein export to the red cell surface (A. Cowman, personal communication). Most of those assays are complex and not amenable to high throughput screening, but they are certainly approachable with the tens of thousands of existing whole-cell hits. It may not be possible to assign individual targets to all compounds, particularly to those hitting more than one target, but validating a given pathway or cellular process as amenable to pharmacological intervention will still be useful. A chemical genomics program to elucidate the mode of action of the published antimalarial hits is possible today but would need the kind of community commitment, leadership, and funding that enabled the functional analysis of model organisms through coordination of tasks and resources and the sharing of data and reagents. We would support such an initiative by catalysing it and as active members of a reagent and information-sharing consortium if it were formed. Participating groups could make use of existing public repositories such as the Malaria Research and Reference Reagent Resource Center (MR4; http://www.mr4.org/). Funding for the fly, worm, and yeast functional genomics efforts was largely justified by their potential contributions to human health. Now we have the tools to identify the drugable genome of the pathogen responsible for the most deadly form of malaria, as defined by currently existing drug-like molecules. In an ideal world such a project should not need additional justification, but we hope the malaria community will rise to the challenge in today's funding environment.
